# Assessing the Muscle–Bone Unit in Girls Exposed to Different Amounts of Impact-Loading Physical Activity—A Cross-Sectional Association Study

**DOI:** 10.3390/children11091099

**Published:** 2024-09-07

**Authors:** Valentina Cavedon, Marco Sandri, Carlo Zancanaro, Chiara Milanese

**Affiliations:** Laboratory of Anthropometry and Body Composition, Department of Neurosciences, Biomedicine and Movement Sciences, University of Verona, 37129 Verona, Italy; valentina.cavedon@univr.it (V.C.); sandri.marco@gmail.com (M.S.); chiara.milanese@univr.it (C.M.)

**Keywords:** dual-energy X-ray absorptiometry, bone mineral content, bone mineral density, fat-free soft tissue mass

## Abstract

Background/Objectives: In children, an association exists between muscle and bone, as well as between physical activity and osteogenesis. Impact loading is a factor in increasing bone accrual during growth. In this work, we explored the muscle–bone association in girls exposed to long-term physical activity at different levels of impact loading. Methods: Four groups of girls aged 7–16 were considered. The curricular (C; *n* = 22) group only had curricular physical activity at school (2 h/w). In addition to curricular physical activity, the girls in the dance (D; *n* = 21), gymnastics at lower training (GL; *n* = 14), and gymnastics at higher training (GH; *n* = 20) groups had 2 h/w, 4 h/w, and 4 h/w < training ≤ 12 h/w additional physical activity, respectively, for at least one year. A visual analysis estimated the respective amounts of impact-loading activity. The bone mineral content (BMC), areal bone mineral density (aBMD), and fat-free soft tissue mass (FFSTM) were assessed with dual-energy X-ray absorptiometry. Results: The results showed that, after adjusting for several confounders, statistically significant correlations were present between muscle mass and several bone mineral variables. A regression analysis confirmed the correlation in the data, and showed the marginal role of other body composition variables and physical activity for predicting BMC and BMD. Conclusion: Skeletal muscle mass is a major determinant of the BMC and BMD of the TBLH, as well as of the Appendicular level, in girls exposed to different amounts of long-term impact-loading physical activity.

## 1. Introduction

As early as 1987, Frost proposed the mechanostat theory [1], suggesting that bone homeostatically adapts to maintain its strength in relation to the strain caused by physiological loads. Muscle contraction is the primary physiological load on bone, beyond gravity and impact-loading movements. During everyday activities, muscles put forces on bones larger than those of gravity, because most muscles move joints using unfavorable lever arms. Based on the mechanostat theory, Frost and Schönau described the muscle–bone unit [2]. The muscle–bone unit can be assessed by evaluating either the ratio of muscle mass to bone mass/density (structural muscle–bone unit), or the ratio of muscle function (strength or its proxy, e.g., the muscle cross-sectional area) to bone mass/density (functional muscle–bone unit), the latter being primarily used in clinical disorders of the bone in children and adolescents [3]. The intensity of the crosstalk between bone and muscle is especially high in the pediatric population and declines (albeit persisting) in adulthood [4]. Therefore, investigating the muscle–bone unit is particularly relevant in the pediatric population.

In children, both muscle and bone mass increase with chronological age because of general body growth [5,6,7]. It is well known that childhood is a crucial period for the accrual of bone mineral [8,9], and achieving a higher peak bone mass is protective against the loss of bone mineral density later in life [10]. Accordingly, maximizing bone mineral accrual in youth is paramount for preventing osteoporosis, especially in females, who are typically more at risk for this pathology than males. While genetic factors are the major determinants of bone mineral density, epigenetic factors, such as nutrition, body composition, and physical activity, contribute to different extents over the lifespan. The lean mass and skeletal muscle mass are positively associated with the bone mineral content (BMC) in Caucasian children and adolescents [11,12]. It has been shown in children that peak lean body mass (a proxy for muscle mass/strength) accrual precedes peak bone mineral content (a proxy for bone mass/strength) accretion [5], and local muscle contractions may improve bone mineral content in the long term [13], thereby supporting the concept that muscle action stimulates increases in bone strength. This concept has been confirmed in a recent review and meta-analysis [14], which showed a statistically significant association between changes in bone mineral density (BMD) and various muscle variables.

Physical activity (either physical exercise or sports participation) positively affects muscle, as well as bone accrual [15,16,17,18,19]. However, the response of bone tissue to physical activity differs according to the degree of impact loading imposed upon the bone [2,20,21], and there is convincing evidence that modes of physical activity involving impact loading have a superior impact on bone vs. non-impact activities [18,22,23,24]. Therefore, investigating the muscle–bone unit from the perspective of optimal bone mineral accrual in childhood and adolescence is of particular significance in children performing physical activity with different amounts/types of impact-loading exercise.

Dance is a popular modality of physical activity all over the world, and can start early in childhood. Dance is predominantly practiced by girls [25], possibly because it is associated with the improvement of posture, as well as physical fitness, motor coordination, mood, and body image. Dance can be practiced at different intensity levels, from recreational to dance sport. Moderate engagement in recreational dance training has shown favorable effects on both body composition and cardiovascular fitness in college-aged females [26,27]. It involves some impact-loading activity [26,27,28,29], suggesting additional positive effects on bone. Similar to dance, artistic gymnastics is especially preferred by young girls [25,30]. It has a well-known positive effect on body fat mass (FM) as well as fat-free mass (especially its bone mineral component) [31,32], showing a stronger effect than other sports [33], in proportion to the amount of participation [34].

As far as we know, the muscle–bone association in children exposed to different amounts of impact-loading physical activity has not been studied yet. In this work, we used dual-energy X-ray absorptiometry (DXA) to characterize the association between muscle and bone in girls exposed to different amounts of impact-loading physical activity over a large age span in the context of other relevant variables, i.e., body mass and size, body composition variables and indices other than skeletal muscle mass, and the amount of physical exercise, as possible contributors to the BMC and BMD. Since a previous work [35] suggested that the bone–muscle relationship can be masked in those with high levels of physical training, we did not consider highly trained girls in this study.

## 2. Materials and Methods

### 2.1. Participants

Advertising and word of mouth were used to recruit a convenience sample of Caucasian girls. An a priori analysis carried out with G* Power showed that a total sample of 68 subjects was required to carry out a multiple regression with alpha = 0.05, power = 0.80, and a medium effect size (Cohen f2) of 0.15. This study complied with the Declaration of Helsinki and was approved by the ethics committee of the Department of Neurosciences Biomedicine and Movement Sciences (Prot. N. 33726/2013). The girls and their parents gave their written, informed consent. The inclusion criteria were the following: 7 y < age <16 y; and stature, body mass, and body mass index (BMI) within the 3rd and 97th percentiles for the reference population of northern–central Italy [36]. For inclusion in the curricular (C) group, regular participation in curricular physical education classes (2 h/w), with no other extracurricular, regular physical activity was required; for inclusion in the dance (D) or gymnastics (G) group, the girls had to be regular participants in curricular physical education classes (2 h/w) as well as recreational dancing (both classic and modern) for a maximum of 2 h/w, or gymnastics for at least one year. The gymnasts were divided into a lower-training (GL, training up to 4 h/w) or higher-training (GH, 4 h/w < training ≤ 12 h/w) group. It has been suggested (Booth and Leese, 2006) [37] that in this type of study, only physically active participants should be included. Accordingly, girls with an MET-min/w score > 600 (see below for an explanation) were included in the analysis. The exclusion criteria were the presence of a concurrent musculoskeletal pathology, injury in the previous six months, amenorrhea (no menses for at least three months), and any ongoing pharmacological treatment.

### 2.2. Collection of Participants’ Characteristics

Menstrual status and age at menarche were determined by questionnaire. Following Ainsworth et al. [38], energy expenditure was estimated in all the girls and expressed as the metabolic equivalent of task (MET-min/w). The MET is calculated as a multiple of the resting metabolic rate [39] and is a currently adopted modality to express the energy cost of physical activities. The number of impact-loading maneuvers in a typical training session was registered [40] and used an estimate of the girls’ impact-loading exposure.

### 2.3. Anthropometry and Body Composition

A Tanita electronic scale (BWB-800 MA, Wunder SA.BI. Srl, Milano, Italy) was used to measure body mass to the nearest 0.1 kg. Stature was measured using a Harpenden stadiometer (Holtain Ltd., Crymych, Pembs, UK) to the nearest mm. The body mass index (BMI) was calculated as weight (kg)/height (m^2^).

The fat mass, fat-free soft tissue mass (FFSTM), BMC, and areal bone mineral density (aBMD) were determined using a total body DXA scanner (QDR Explorer W, Hologic, Marlborough, MA, USA; fan-bean technology, software for Windows XP version 12.6.1). The reference phantom supplied by the manufacturer was used for the daily control of possible baseline drift. The same operator carried out all the analyses to ensure consistency. DXA scanning was performed late in the morning in a post-absorptive state. The participants were invited to avoid intense exercise for the preceding 24 h. The scans were performed with participants wearing lightweight clothing with no metal accessories. All the measurements were performed according to the manufacturer’s protocol. Scans were taken of the whole body (WB) and lumbar spine (L1–L4) in the anteroposterior projection, according to current indications for children and adolescents [41]. The left Ward’s triangle at the hip and the right forearm (33% radius/ulna, also called the 1/3 radius/ulna, primarily formed of cortical bone; ultradistal region, consisting mainly of trabecular bone) were also examined as relevant target sites for physical activity [42,43,44,45]. Velcro restraints were applied around the participants’ ankles during the WB scan to avoid movement. During the WB scan, the examination of Ward’s triangle, and lumbar spine scanning, the participants were placed supine; for the forearm measurements, the participants were seated. The in vivo, short-term precision was calculated by the repeated (*n* = 3) scanning of 15 subjects with repositioning (International Society for Clinical Densitometry, http://www.iscd.org/, accessed on 12 February 2024). In vivo precision was not assessed in the participants of the present study because of multiple radiation exposures and the recognized precision of DXA. For the WB DXA measurements, the in vivo short-term precision was 2.3%, 2.8%, 0.5%, 1.14, and 0.9% for FM, %FM, FFSTM, BMC, and BMD, respectively. The precision was 1.43%, 0.71%, and 3.74%, and 1.28%, 0.98%, and 1.27%, for the lumbar spine (L1–L4), total radius and ulna, and total hip BMC and BMD, respectively. The Hologic software (version 12.6.1) readings divided the WB scans into the trunk, entire arm (left and right), entire leg (left and right), and head. The total body less head (TBLH) region BMC and aBMD were used in the analysis because the skull contains a large fraction of the total body minerals [46], which is not sensitive to physical activity [47].

The Appendicular (sum of the arms and legs) FM and FFSTM were also calculated. The Appendicular FFSTM is a reliable proxy for skeletal muscle mass [48] and is a current parameter with which to assess the muscle–bone relationship; accordingly, the Appendicular FFSTM was chosen as the primary predictor variable with which to model the muscle–bone relationship in the regression analysis. The FM and FFSTM were also normalized by stature, by calculating the FM index (FMI) and FFSTM index (FFSTMI) [49,50,51,52]. Indices were calculated for the whole body (WB) and the Appendicular region by dividing the DXA-derived FM (in kg) and FFSTM (in kg) by the squared stature (in meters).

### 2.4. Statistical Analysis

The normality of the data was assessed using the Kolmogorov–Smirnov test. The continuous variables are expressed as the mean ± SD, while the categorical data are shown as frequencies and percentages. Group means were compared with ANOVA. A post hoc analysis was conducted using the Bonferroni correction when homogeneity of variance was present, and the Games–Howell correction when it was absent, as determined by the Levene test.

The association between body composition variables and bone mineral variables were investigated using a sequential approach. First, the Pearson’s r was used to assess the bivariate correlation between the variables. The strength of the correlation was rated following Hopkins [53]: small (0–0.30), moderate (0.31–0.49), large (0.50–0.69), very large (0.70–0.89), and almost perfect (0.90–1). A partial correlation analysis (r_PC_) was used to evaluate the association between the Appendicular FFSTM and bone variables, controlling for the effects of body mass, stature, age in months, group (C, D, GL, GH), and MET-min/w. Second, the Appendicular FFSTM and individual bone mineral variables were used as the independent and dependent variables, respectively, in a regression analysis. The goodness of fit of the regression models was assessed by calculating the coefficient of determination R^2^ and the standard error of the estimate (SEE). Third, a joint multivariable analysis was carried out to identify the demographic and body composition variables (including the Appendicular FFSTM) predictive of bone mineral variables. An analysis was performed by estimating the permutation variable importance measures (VIMs) using random forests [54]. VIMs measure the difference in the distribution of each variable between groups individually, and in multivariate interactions with other variables. The confidence intervals (CIs) of the VIMs were calculated with the method of Ishwaran and Lu [55], and the subset of variables with CIs not intersecting the zero line was selected. The selected variables were entered as independent variables in the regression analysis.

The statistical analyses were performed using IBM-SPSS v.25 (IBM Corp., Armonk, NY, USA) and R 4.4.0 (R Core Team, Vienna, Austria). A *p* value ≤ 0.05 was considered statistically significant.

## 3. Results

Seventy-seven physically active girls aged 7–16 participated in this study; therefore, according to the a priori analysis, this study was sufficiently powered. The mean demographic characteristics of the sample were as follows: age, 134.8 ± 25.97 months; body mass, 38.8 ± 11.98 kg; stature, 144.6 ± 11.44 cm; BMI, 18.1 ± 2.86 kg/m^2^; age at menarche, 11.6 ± 0.45 y (*n* = 31). The girls in the D, GL, and GH groups had been practicing the respective impact-loading physical activity for a mean of 3.0 ± 2.73 y. The demographic characteristics and energy expenditure of the four groups (C, D, GL, and GH) are summarized in Table 1.

The recreational dancers aged 7–11 had an average of 450 low-impact and 200 high-impact loading movements per week. The dancers aged 12–15 had 800 and 230 low-impact and high-impact loading movements, respectively. Girls in the GL group averaged 562/w and 964/w repetitions of low- and high-impact loading movements, respectively. The corresponding figures for the girls in the GH group were 1288/w and 1932/w, and 2456/w and 3684/w, respectively. Accordingly, the exposure to high impact loading movements in the GL and GH groups was about five and ten times that of the D group, respectively.

A one-way ANOVA showed that the four groups were not statistically significantly different in their age in months (F = 0.640, *p* = 0.593), body mass (F = 2.420, *p* = 0.073), or BMI (F = 0.671, *p* = 0.573). A statistically significant difference was found for stature (F = 4.017, *p* = 0.011). According to a post hoc analysis, stature was higher in C vs. GL (*p* = 0.045) and, at the limit of statistical significance, GH (*p* = 0.053). The figure for the MET-min/w was about 1000 in the C group, showing that the girls therein were physically active. The MET-min/w showed a statistically significant difference among the four groups (F = 31.482, *p* <0.001). According to the post hoc analysis, the MET-min/w was statistically significantly higher in GL and GH vs. C (*p* < 0.001 for both) and D (*p* = 0.014 and *p* < 0.001, respectively); the MET-min/w was higher in GH vs. GL (*p* = 0.003).

Table 2 shows the mean values of the Appendicular FFSTM and FFMI, as well as the WB fat and FMI for the four groups of girls.

A one-way ANOVA showed that the Appendicular FFSTM was not significantly different in the four groups (F = 0.718, *p* = 0.544). The Appendicular FFMI was different in the four groups at the limit of statistical significance (F = 2.257, *p* = 0.043); a post hoc analysis showed that the FFMI was higher in GH vs. C (*p* = 0.039). Both the WB FM and WB FMI were statistically significantly different in the four groups of girls (F = 8.500, *p* < 0.001; F = 8.480, *p* < 0.001); a post hoc analysis showed that the WB FM was lower in GH and GL vs. C (*p* <0.001; *p* = 0.009, respectively) and in GH vs. D (*p* = 0.004) and, at the limit of statistical significance, in GL vs. D (*p* = 0.067). The WB FMI was lower in both GH and GL vs. C (*p* < 0.001 and *p* = 0.010, respectively) and D (*p* = 0.03 and *p* = 0.05, respectively).

**Figure 1:**
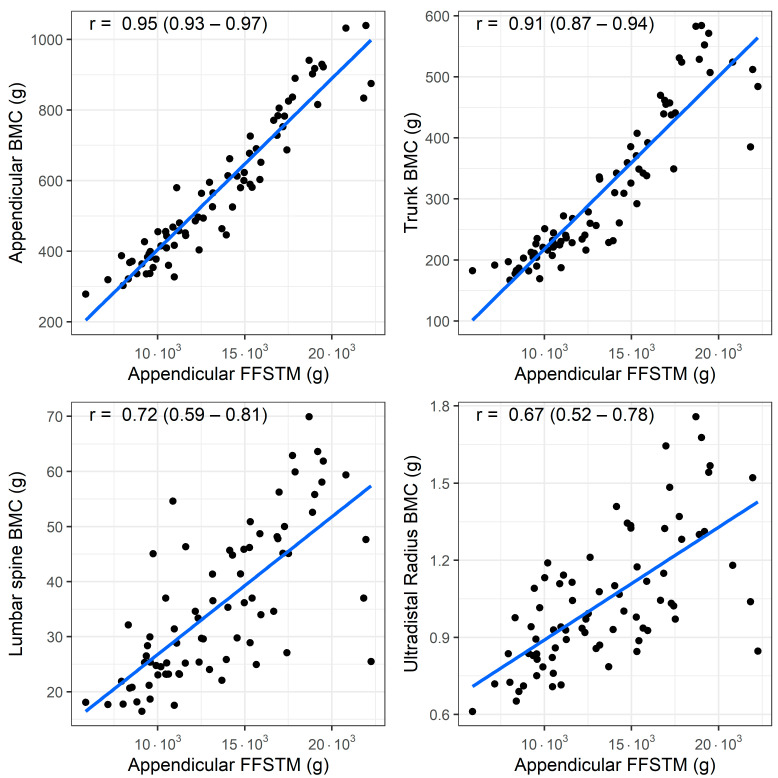
Association (Pearson’s r) between fat-free soft tissue mass (FFSTM) and bone mineral content (BMC) at four sites.

**Figure 2:**
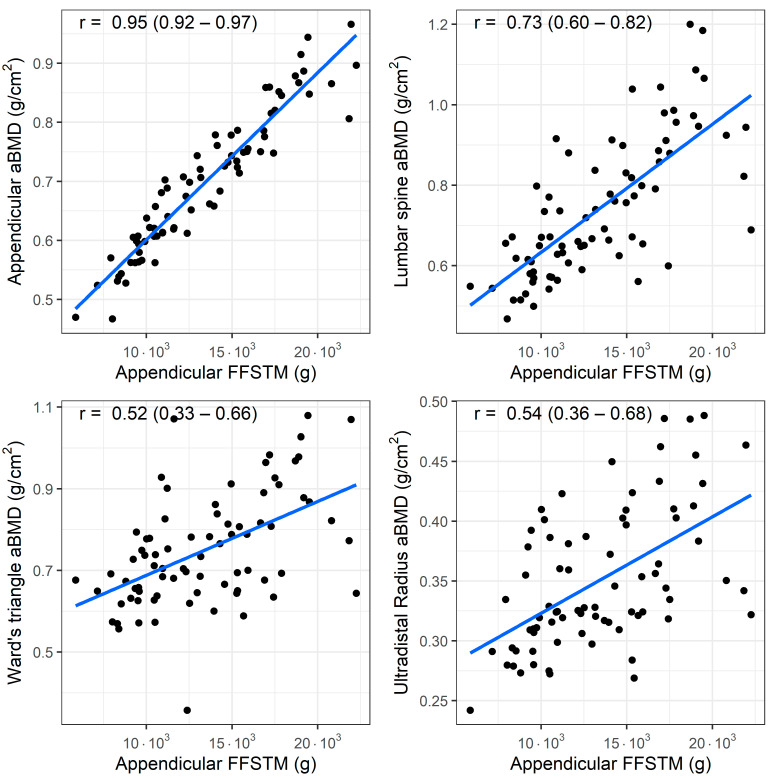
Association (Pearson’s r) between fat-free soft tissue mass (FFSTM) and areal bone mineral content (aBMD) at four sites.

A bivariate correlation analysis (*n* = 77) showed a statistically significant association between the Appendicular FFSTM and several bone mineral variables. The relationship between the Appendicular FFSTM and selected BMC and aBMD variables is presented in Figure 1 and Figure 2, respectively.

The strength of the correlation with the Appendicular FFSTM is as follows: it is almost perfect for Appendicular BMC (r = 0.95, *p* < 0.001), TBLH BMC (r = 0.94, *p* < 0.001), trunk BMC (r = 0.91, *p* < 0.001), TBLH aBMD (r = 0.93, *p* < 0.001), and Appendicular aBMD (r = 0.91, *p* < 0.001); very large for pelvis BMC (r = 0.89, *p* < 0.001), pelvis aBMD (r = 0.88, *p* < 0.001), lumbar spine BMC (r = 0.71, *p* <0.001), and lumbar spine aBMD (r = 0.73, *p* < 0.001); large for Ward’s triangle aBMD (r = 0.51, *p* < 0.001), and ultradistal radius BMC and aBMD (r = 0.66 and r = 0.54, *p* < 0.001 for both); and moderate for Ward’s triangle BMC (r = 0.50, *p* < 0.001). No statistically significant correlation was found between Appendicular FFSTM and trunk BMD (r = 0.12, *p* = 0.294).

Partial correlation analysis adjusting for age, stature, body mass, menarche, MET-min/w, and group (C, D, GL, GH) showed that Appendicular FFSTM correlated with TBLH BMC and BMD (r_(PC)_ = 0.31, *p* = 0.008; r_(PC)_ = 0.39, *p* = 0.001, respectively), Appendicular BMC and BMD (r_(PC)_ = 0.36, *p* = 0.002; r_(PC)_ = 0.45, *p* < 0.001, respectively), and pelvis BMC (r_(PC)_ = 0.31, *p* = 0.010).

A linear regression analysis using the Appendicular FFSTM as the predictor yielded statistically significant models for most of the bone mineral variables (Table 3). The R2 values were highest (>0.75) for TBLH BMC, TBLH aBMD, Appendicular BMC, Appendicular aBMD, pelvis BMC, pelvis aBMD, and trunk BMC. For the lumbar spine BMC and BMD, Ward’s triangle BMC and BMD, and ultradistal radius BMC and aBMD, the R2 ranged from 0.058 to 0.52 (*p* range, from 0.019 to <0.001). The Appendicular FFSTM did not significantly predict the trunk aBMD.

**Figure 3:**
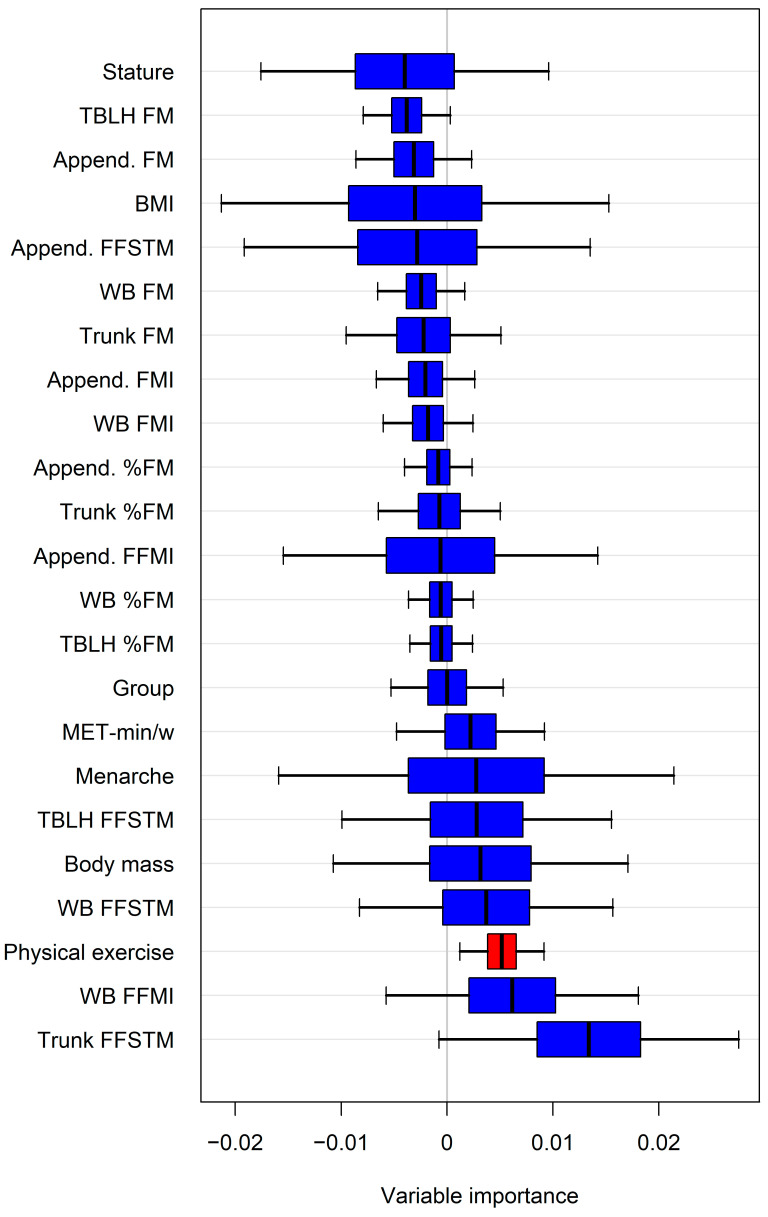
Representative example of variable importance estimated by random forests for predicting lumbar spine aBMD. Only variable(s) with confidence intervals not intersecting zero line were selected for regression analysis. For abbreviations, see text. Append., Appendicular.

The variable importance estimated by random forests (representative example in Figure 3) identified additional potential predictors of bone mineral variables. When these predictors were introduced individually into the regression model alongside the Appendicular FFSTM, a statistically significant increase in R^2^ and a reduction in the SEE were observed for several variables (Table 4).

Specifically, physical activity improved the model for ten bone mineral variables, the WB FFMI for seven, the trunk FFSTM for six, and the body mass for two. However, in three cases, adding the variable selected by the random forests did not significantly improve R^2^ (WB FFMI for Appendicular aBMD; physical activity for pelvis aBMD and lumbar spine aBMD).

## 4. Discussion

In this cross-sectional study, we investigated the association between muscle and bone in girls aged 7–16 exposed to curricular physical activity alone or curricular physical activity plus different amounts of long-term (mean practice, 3.0 ± 2.73 y) impact-loading physical activity using DXA. The additional impact loading was acquired through popular leisure activities, such as dance and gymnastics, at different levels of participation. The four groups of girls (C, D, GL, and GH) were not statistically significantly different for age in months, body mass, BMI (Table 1), or Appendicular FFSTM (Table 2). The results show that the four study groups were well balanced in terms of age and BMI at the baseline. This balance is crucial, as it minimizes the risk of confounding bias, ensuring that any observed effects can be more confidently attributed to the interventions rather than to differences in demographic or anthropometric characteristics. Nevertheless, as expected, the four groups differed significantly in energy consumption (MET-min/w) due to their varying levels of physical activity.

The results highlight three key findings: (a) a statistically significant association is present between muscle mass and various bone mineral variables in girls aged 7–16, regardless of age, body mass, stature, or energy expenditure; (b) the DXA-derived Appendicular FFSTM is a significant predictor of several bone mineral variables; and (c) this predictive capability can be marginally enhanced by incorporating additional body composition variables or physical activity levels.

The results presented in this paper confirm and extend previous data that have shown a positive association between skeletal muscle and/or physical activity and bone characteristics in children (reviewed in [12,14,56]). The correlations between the Appendicular FFSTM (a proxy of skeletal muscle mass) and the BMC and aBMD were positive and ranged from almost perfect (r ≥ 0.90), to very large (0.70 < r < 0.89), large (0.50 < r < 0.69), and moderate (0.30 < r < 0.49) for the TBLH and most sites (see Results), with *p* < 0.001 for all correlations. These data clearly indicate the tendency of the bone mineral content and areal density to increase with increasing amounts of muscle mass in the sample, in accordance with previous findings on children and adolescents [57], especially females [58]. At the TBLH- and Appendicular level, such an association was robust enough when adjusted for several confounding variables (age in months, stature, body mass, menarche, and energy expenditure), albeit at a lower strength. Taken together, these results suggest that, in our sample, the muscle–bone unit was largely independent of the impact-loading activity. Similarly, Baptista et al. [59] showed that lean mass is the most important predictor of bone in girls, while habitual physical activity is not. At the regional level (lumbar spine, Ward’s triangle, ultradistal radius), the correlation between the Appendicular FFSTM and BMC and aBMD was no longer statistically significant after adjusting for confounders, indicating that local factors could be involved in mediating the effect of physical activity and/or impact loading on bone [60,61,62,63].

To further explore the relationship between skeletal muscle and bone variables, the Appendicular FFSTM was used in a regression analysis as the predictor of the BMC and aBMD. The results (Table 3) showed that the skeletal muscle mass predicts all the bone variables effectively except for the trunk aBMD (*p* value for beta coefficient < 0.001 for all) and small SEE. However, the coefficient of determination (i.e., the proportion of variance in the dependent variable that is predictable from the independent variable) was much higher at the TBLH and Appendicular BMC and BMD level (R^2^ range from 0.867 to 0.906) than the regional level (lumbar spine, Ward’s triangle, ultradistal radius; R^2^ range from 0.238 to 0.522), thereby confirming the decreasing ability of the skeletal muscle mass to determine the BMC and aBMD when approaching the regional level.

It has been previously shown that variables other than skeletal muscle mass (e.g., fat mass) may affect the BMC and aBMD in children [64]. To explore this possibility, we selected the most potentially predictive variables of the BMC and aBMD through a random forest approach, as typified in Figure 3. Forcing each random forests selected variable into the regression model run with the Appendicular FFSTM as the predictor showed that some body composition variables, as well as the amount of physical activity, yielded a statistically significant increase in R^2^ (and a parallel decrease in the SEE) for several bone variables (Table 3). In particular, the greatest, statistically significant changes in R2 were associated with the amount of physical activity, with the regional bone variables being especially affected (lumbar spine, trunk and Ward’s triangle BMD, and Ward’s triangle BMC). These data are supported by previous findings in girls that have shown that impact-loading activity may be more effective for increasing BMD at the regional than WB level [65,66,67,68]. Lower, albeit statistically significant, changes in R^2^ were associated with the trunk FFSTM and WB FFMI, with the affected variables being the trunk BMC or trunk sub-regional variables (lumbar spine BMC, pelvis BMC, and aBMD). In accordance with this, a moderate (r = 0.36), statistically significant correlation was found between the total trunk muscle mass (evaluated using a CT) and spinal BMD in premenopausal Korean females (Kang et al., 2016) [69]. In this study, the inherent limitation of DXA technology in differentiating between muscle mass and visceral mass in the trunk prevented the confirmation of such a finding in our sample. Overall, these data suggest that the muscle–bone unit is positively affected by body weight loading at the trunk level. The FFMI (also known as the Skeletal Mass Index, SMI) provides information on body compartments regardless of stature. The FFMI has been shown to be positively correlated with the BMC and/or BMD at the WB and regional levels in adults [70] and in young adults of both sexes. Herein, we showed that the WB FFMI improves the predictive power of the Appendicular FFSTM for the trunk and pelvis BMC, as well as the pelvis BMD. Since the Appendicular FFSTM is a substantial part of the WB FFSTM, we argue that the additional predictive power is associated with body weight loading at the trunk level.

The present work has some limitations that should be acknowledged. First, it was a cross-sectional study, not longitudinal, which prevents us from making causal inferences. Second, we were unable to gather information on the habitual diet of all the participants, meaning we could not assess calcium intake—a crucial factor in bone mineralization. Third, we did not measure muscle strength, which means we could not investigate the muscle–bone unit from a functional point of view. This work also has some strengths. First, we used the gold standard for bone mineral assessment, i.e., the DXA. Second, we recruited girls over a large age interval (ten years), including both pre- and postmenarcheal participants, thereby exploring a crucial period in bone mineralization. Third, we used the fractional age in the analysis, ensuring the fine-tuning of the effect of chronological age on the bone variables.

## 5. Conclusions

In conclusion, the results of this work show that skeletal muscle mass is a major determinant of bone mineral at the TBLH and Appendicular levels in girls exposed to different amounts of long-term impact-loading physical activity. Further work investigating skeletal muscle quality is needed to clarify the role of different amounts of impact-loading physical activity on bone mineral, especially at the local level. The results presented herein are of use to coaches and healthcare providers insofar as they underline the relevance of muscle mass accrual to bone health in growing girls.

## Figures and Tables

**Table 1:** Demographic characteristics and energy expenditure of the four groups of girls. Means ± SD.

Variable	Group			
	C (*n* = 22)	D (*n* = 21)	GL (*n* = 14)	GH (*n* = 20)
Age (months)	141.5 ± 25.30	136.7 ± 26.29	128.8 ± 25.10	134.0 ± 33.22
Body mass (kg)	42.3 ± 12.47	40.9 ± 11.98	33.8 ± 9.90	35.2 ± 11.18
Stature (cm)	149.7 ± 12.91	147.2 ± 12.77	137.6 ± 12.04 *	139.0 ±13.77
BMI (kg/m^2^)	18.4 ± 3.04	18.5 ± 3.25	17.5 ± 2.15	17.7 ± 2.24
MET-min/w	1090.9 ± 596.63	1357.5 ± 805.32	2083.6 ± 365.90 *^	2938.7 ± 740.55 *^§

C, curricular physical activity only; D, curricular physical activity plus dance; GL, curricular physical activity plus gymnastics at lower training volume; GH, curricular physical activity plus gymnastics at higher training volume; BMI, body mass index; MET, metabolic equivalent of task. *, *p* < 0.05 vs. C; ^, *p* < 0.05 vs. D; §, *p* < 0.05 vs. GL.

**Table 2:** Appendicular FFSTM and WB FM, and their respective stature-adjusted indices FFMI and FMI, for four groups of girls. Means ± SD.

Variable	Group			
	C (*n* = 22)	D (*n* = 21)	GL (*n* = 14)	GH (*n* = 20)
Appendicular FFSTM (g)	12,450.7 ± 3561.82	12,366.3 ± 3348.86	10,791.7 ± 2938.54	12,103.6 ± 4233.81
Appendicular FFMI (kg/m^2^)	6.0 ± 0.89	6.2 ± 0.80	6.3 ± 0.62	6.8 * ± 1.10
WB FM (g)	11,368.6 ± 4627.86	10,498.9 ± 4460.86	7078.6 ± 3073.31 *	6284.9 ± 2147.77 *^
WB FMI (kg/m^2^)	11.4 ± 4.63	10.5 ± 4.46	7.1 ± 3.07 *^	6.3 ± 2.15 *^

C, curricular physical activity only; D, curricular physical activity plus dance; GL, curricular physical activity plus gymnastics at lower training volume; GH, curricular physical activity plus gymnastics at higher training volume; FFSTM, fat-free soft tissue mass; FFMI, fat-free mass index; WB, whole body; FMI, fat mass index. *, statistically significant difference vs. C; ^, statistically significant difference vs. D.

**Table 3:** Results of regression analysis using Appendicular FFSTM as predictor (*n* = 77).

Variable	Adjusted R^2^	Beta Coefficient	t Value	*p* Value	SEE
TBLH BMC (g)	0.892	0.945	25.066	<0.001	105.0
Appendicular BMC (g)	0.906	0.953	27.088	<0.001	61.4
Trunk BMC (g)	0.827	0.911	19.074	<0.001	50.8
Pelvis BMC (g)	0.788	0.889	16.820	<0.001	25.9
Lumbar spine BMC (g)	0.504	0.715	8.850	<0.001	9.7
Ward’s triangle BMC (g)	0.238	0.498	4.974	<0.001	0.16
Ultradistal radius BMC (g)	0.434	0.664	7.696	<0.001	0.19
TBLH aBMD (g/cm^2^)	0.867	0.932	22.247	<0.001	0.0422
Appendicular aBMD (g/cm^2^)	0.891	0.945	24.976	<0.001	0.0391
Trunk aBMD (g/cm^2^)	0.002	0.121	1.057	0.294	0.3485
Pelvis aBMD (g/cm^2^)	0.778	0.884	16.371	<0.001	0.0842
Lumbar spine aBMD (g/cm^2^)	0.522	0.727	9.172	<0.001	0.1188
Ward’s triangle aBMD (g/cm^2^)	0.252	0.511	5.154	<0.001	0.119
Ultradistal radius aBMD (g/cm^2^)	0.281	0.539	5.547	<0.001	0.0495

FFSTM, fat-free soft tissue mass; CI, confidence interval; SEE, standard error of the estimate; R^2^, adjusted coefficient of determination; TBLH, total body less head; BMC, bone mineral content; aBMD, areal bone mineral density. The SEE is in the same units as the predicted variable.

**Table 4:** Effect of entering random forests selected variable in regression models using Appendicular FFSTM as predictor.

Additional Predictor	Predicted Variable	R^2^	Change in R^2^	*p* Value of R^2^ Change	SEE	Change in SEE
Body mass (kg)	TBLH BMC (g)	0.899	+0.007	0.013	101.3	−3.7
	App BMC (g)	0.914	+0.008	0.006	58.7	−2.7
WB FFMI (kg/m^2^)	TBLH BMC (g)	0.921	+0.029	<0.001	90.0	−15.0
	Appendicular BMC (g)	0.923	+0.017	<0.001	55.5	−5.9
	Trunk BMC (g)	0.878	+0.051	<0.001	42.6	−8.2
	Pelvis BMC (g)	0.809	+0.021	0.003	24.6	−1.3
	TBLH aBMD (g/cm^2^)	0.876	+0.009	0.013	0.041	−0.0037
	Appendicular aBMD (g/cm^2^)	0.897	+0.006	0.076	0.0385	−0.0006
	Pelvis aBMD (g/cm^2^)	0.818	+0.040	<0.001	0.0764	−0.0078
Trunk FFSTM (kg/m^2^)	Trunk BMC	0.885	+0.058	<0.001	41.3	−9.5
	Pelvis BMC	0.817	+0.029	0.001	24.1	−1.8
	Lumbar spine BMC	0.566	+0.062	0.001	9.0	−0.7
	TBLH aBMD (g/cm^2^)	0.879	+0.012	0.005	0.0405	−0.0017
	Appendicular aBMD (g/cm^2^)	0.896	+0.005	0.043	0.0383	−0.0008
	Pelvis aBMD (g/cm^2^)	0.824	+0.046	<0.001	0.0751	−0.0091
Physical activity (h)	Pelvis BMC	0.798	+0.010	0.031	25.3	−0.6
	Ward’s triangle BMC	0.343	+0.105	0.001	0.15	−0.10
	Ultradistal radius BMC	0.473	+0.003	0.012	0.19	−0.006
	TBLH aBMD (g/cm^2^)	0.887	+0.020	<0.001	0.039	−0.003
	Appendicular aBMD (g/cm^2^)	0.908	+0.017	0.001	0.0364	−0.0027
	Trunk aBMD (g/cm^2^)	0.119	+0.117	0.004	0.3310	−0.0175
	Pelvis aBMD (g/cm^2^)	0.785	+0.007	0.077	0.0830	−0.0012
	Ward’s triangle aBMD (g/cm^2^)	0.337	+0.085	0.002	0.112	−0.007
	Ultradistal radius aBMD (g/cm^2^)	0.355	+0.054	0.003	0.0469	−0.0026
	Lumbar spine aBMD (g/cm^2^)	0.527	+0.257	0.185	0.118	−0.055

FFSTM, fat-free soft tissue mass; R^2^, adjusted coefficient of determination; SEE, standard error of the estimate; TBLH, total body less head; BMC, bone mineral content; aBMD, areal bone mineral density. SEE is in same units as predicted variable.

## Data Availability

Data are available from the corresponding author upon any reasonable request due to privacy reason).
